# P19 A service evaluation to assess antimicrobial resistance in penicillin allergy

**DOI:** 10.1093/jacamr/dlac053.019

**Published:** 2022-05-31

**Authors:** S Ahmed, S Relton, R West, J Sandoe

**Affiliations:** 1 University of Leeds, Leeds, UK; 2 Leeds Teaching Hospitals NHS Trust, Leeds, UK

## Abstract

**Background:**

Antibiotic consumption is one of the main drivers for antimicrobial resistance (AMR). To improve clinical outcomes and tackle AMR, we need to improve antibiotic usage in patients with penicillin allergy (PenA). Patients with PenA have been found to have higher antibiotic usage, often requiring repeated courses of antibiotics compared with those without PenA. Additionally, patients with PenA are more likely to receive broad spectrum antibiotics.

**Objectives:**

To determine whether patients who have PenA are more likely to be colonized or infected with resistant bacteria.

**Methods:**

Data were extracted from the pathology reporting system (Telepath) for blood culture specimens isolating *Staphylococcus aureus* and *Streptococcus pneumoniae* between 2017 and 2019 at Leeds Teaching Hospitals NHS Trust. Sputum samples from 2019 that isolated *S. pneumoniae* and *Haemophilus influenzae* were also analysed. These organisms/samples were chosen as these would indicate the presence of an infection where a penicillin would normally be used as first-line therapy. Where patients had multiple samples only the first sample obtained during the study period was included in the analysis. Standard descriptive statistics was used to summarize the data and characteristics were cross tabulated with penicillin allergy status.

**Results:**

*S. pneumoniae isolated from blood cultures:* A total of 297 patients were included in the analysis, 33/297 (11.1%) had PenA, 50.8% were female and the median age was 60 years (IQR 37–75). Susceptibility results are summarized in Figure 1. Resistant isolates are comprised of isolates with resistant or intermediate susceptibility. *S. aureus isolated from blood cultures:* Analysis included 783 isolates from first patient encounters, 97/783 (12.4%) patients had PenA, 35.1% were female and the median age was 58 years (IQR 39–74.5). Susceptibility results are summarized in Figure 2. *S. pneumoniae isolated from sputum:* A total of 156 isolates were included in the final analysis: 29/156 (18.6%) patients had PenA, 53.3% were female and the median age was 67 years. Susceptibility results are summarized in Figure 3. *H. influenzae isolated from sputum:* A total 719 samples were included, 122/719 (15.8%) patients had PenA, 55.9% were female and median age was 67 years. Susceptibility results are summarized in Figure 4.

**Conclusions:**

PenA prevalence differs across different patient populations. A higher proportion of patients with PenA had resistance in *S. pneumoniae* sputum isolates compared with those without, however this finding did not meet statistical significance. Further studies evaluating the impact of PenA on AMR need to be conducted.

**Figure 1.**
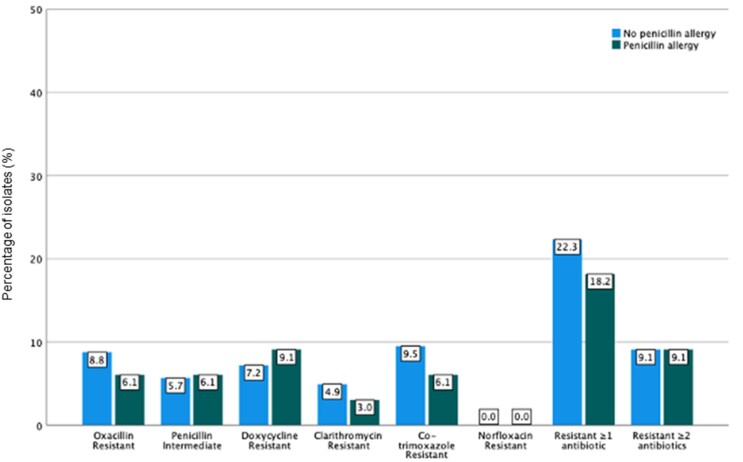
Resistance profile of *S. pneumoniae* isolated from blood cultures according to penicillin allergy status.

**Figure 2.**
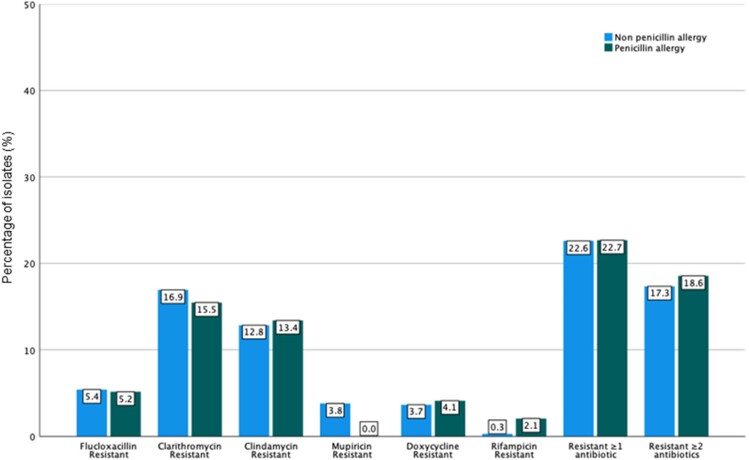
Resistance profile of *S. aureus* isolated from blood cultures according to penicillin allergy status.

**Figure 3.**
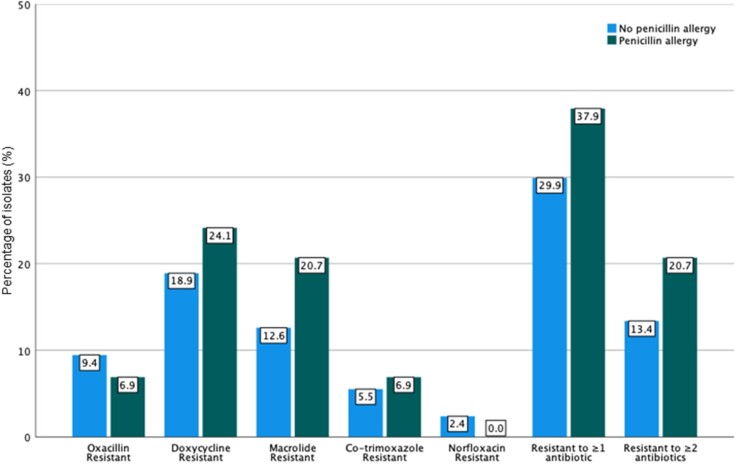
Resistance profile of *S. pneumoniae* isolated from sputum according to penicillin allergy status.

**Figure 4.**
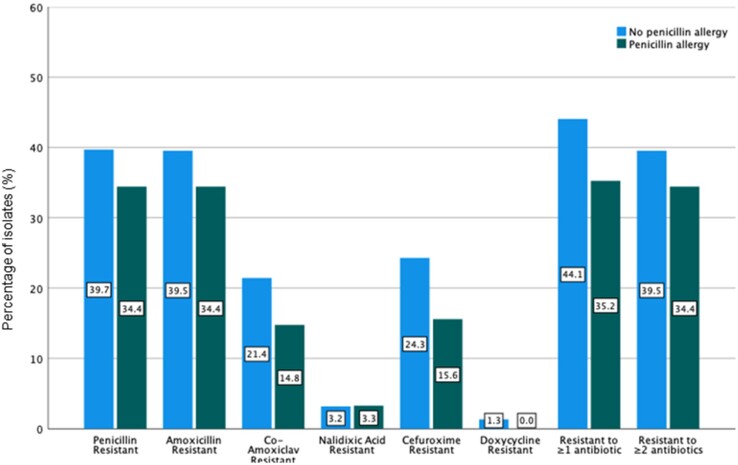
Resistance profile of *H. influenzae* isolated from sputum according to penicillin allergy status.

